# Successful treatment with Cinryze® replacement therapy of a pregnant patient with hereditary angioedema: a case report

**DOI:** 10.1186/s13256-020-02622-3

**Published:** 2021-01-24

**Authors:** Željka Kardum, Višnja Prus, Jasminka Milas Ahić, Darjan Kardum

**Affiliations:** 1 Department of Rheumatology, Clinical Immunology and Allergology, University Hospital Osijek, J. Huttlera 4, 31000 Osijek, Croatia; 2 School of Medicine, Josip Juraj Strossmayer University of Osijek J, Huttlera 4, 31000 Osijek, Croatia; 3 Neonatal Intensive Care Unit, Department of Pediatrics, University Hospital Osijek, J. Huttlera 4, 31000 Osijek, Croatia

**Keywords:** Hereditary angioedema, Pregnancy, C1 replacement therapy, Cinryze®, Case report

## Abstract

**Background:**

Hereditary angioedema (HAE) is a rare disease characterized with recurrent swelling of subcutaneous or mucosal tissue that resolves in approximately 3 days. It can be presented with peripheral edema, abdominal and life-threatening laryngeal angioedema. A variety of triggers are known to cause episodes of angioedema including estrogen exposure. There are different reports regarding the effect of pregnancy on HAE attacks, and in some patients, the pregnancy is a recognized triggering factor.

**Case presentation:**

We present a female Caucasian patient with pre-existing HAE and disease exacerbations during pregnancy, requiring prophylactic use of plasma-derived C1 inhibitor concentrate. She was treated with Cinryze® replacement therapy throughout the pregnancy 1000 IU i.v. 48 times. She gave birth to a healthy male infant, via C-section. After the delivery, the patient was symptom-free for 6 months and required no treatment for HAE.

**Conclusions:**

In the case presented, the angioedema attacks worsened as the pregnancy progressed. The treatment with Cinryze® replacement therapy was effective and safe during pregnancy, with no adverse effects on the infant.

## Introduction

Hereditary angioedema (HAE) is a rare disease characterized with recurrent swelling of subcutaneous or mucosal tissue, without pruritus or urticaria, that resolves in approximately 3 days. The disease can be presented with peripheral edema, abdominal, but also with life-threatening laryngeal angioedema [1].

Several types of HAE are described; the most explored one is due to C1 inhibitor deficiency, known as type I. It is caused by SERPING 1 mutation, on the long arm of chromosome 11, leading to C1 inhibitor deficiency which subsequently results in low C1 inhibitor and C4 protein levels in blood serum [2].

A variety of triggers, such as stress, surgical procedures, some medications (ACE inhibitors, estrogen-containing medication) are identified to cause episodes of angioedema [3, 4]. There are different reports regarding the effect of pregnancy on HAE attacks, and in some patients, the pregnancy is a known triggering factor.

We present a female patient with HAE and frequent disease exacerbations during pregnancy, that required prophylactic use of plasma-derived C1 inhibitor concentrate (pdC1) throughout the pregnancy.

## Case presentation

We present a female Caucasian patient with HAE type I, diagnosed at the age of 26, with SERPING 1 mutation (confirmed by molecular testing), with very low C1inhibitor and C4 levels. Molecular testing was also positive for her father and brother. They also had low C1 inhibitor and C4 levels, but they never had any HAE symptoms.

Her symptoms of HAE first appeared when she was 15 years old, as swelling of her hand, without pain or urticaria. She was misdiagnosed with allergy-induced angioedema in the emergency department and was treated with glucocorticoids and antihistamines, without improvement. Edema subsided 3 days later. After that attack, peripheral angioedema appeared approximately once a year and lasted for a few days.

Since the age of 20 until the age of 26, she had several times very intense abdominal pain, with swelling, that required a visit to the emergency room (ER), where she would receive analgetic and proton -pump inhibitor (PPI) i.v., Gastroenterologist examined her, and after gastroscopy, the diagnosis of chronic erosive gastritis was established. Finally, at the age of 25, due to recurrent peripheral oedema, she was referred to clinical immunologist and allergist. After an extensive workup, at the age of 26, HAE type I was diagnosed.

Once HAE was diagnosed, she was treated with tranexamic acid (1000 mg daily orally) and icatibant 30 mg subcutaneously as on-demand therapy, which she had to use approximately once a year, due to abdominal angioedema.

In her medical history, at the age of 28, she had one spontaneous abortion at 8 weeks of pregnancy. Since the patient planned pregnancy, tranexamic acid was excluded, and recombinant C1 inhibitor, conestat alpha, was provided as on-demand therapy.

At the age of 29, in July 2018. patient came to the emergency room (ER) because of the swelling of her eyelids and lips, and she was treated with a total of 160 mg of methylprednisolone and chloropyramine 20 mg i.v. As she reported difficulty swallowing, an examination by ear, nose and throat (ENT) specialist was performed, and the edema of the epiglottis and sinus piriformis was established. Since there was no improvement after the anti-allergic therapy, recombinant C1 inhibitor was applied, (conestat alpha 2100 IU i.v.) and total regression of edema ensued a few hours later. She was admitted to the Department of Rheumatology, Clinical Immunology and Allergology for further observation.

Her social history revealed that she is a tradeswoman by profession, but is currently unemployed, married, living in a family house with her husband in a rural area, without any domestic animals in her household. The patient is a nonsmoker; she denied using alcohol, drugs or other medications. On admission, her temperature was 36.8°C, heart rate 76 beats/min, blood pressure 120/75 mmHg. There was no swelling of her face, neck or uvula. Her chest was clear to auscultation bilaterally, no wheezing. S1S2 were heard, no murmur, rubs or gallops. The abdomen was not distended; there was no tenderness on palpation and no organomegaly. There was no swelling or edema of her extremities. Neurological examination was unremarkable, with no nuchal rigidity, ophthalmic abnormalities, or cranial nerve signs.

During hospitalization, pregnancy was confirmed (8 weeks gestation). According to HAE guidelines, a pdC1 inhibitor is recommended in pregnancy, and Cinryze® was  advised as only available pd1C1 inhibitor in our country at that time. In July 2018, she received the first application of Cinryze® 1000 IU i.v. and it was prescribed as on-demand therapy.

Two weeks later, she returned to the ER because of nausea and intense abdominal pain. Examination by obstetrics and gynaecology (OBGYN) specialist was performed, and complications related to pregnancy were excluded. The ultrasound of the abdomen displayed a small amount of fluid around the liver, spleen and intestines, and her blood workup was unremarkable.

After surgical and gastroenterologist (GE) examination she was referred to clinical immunologist and abdominal angioedema attack was diagnosed. She received Cinryze® 1000 iu i.v. and afterwards admitted to the Department for further evaluation. Blood workup revealed mild leukocytosis 14 ×109/L (ref. 3.4–9.7 × 109/L), normal C-reactive protein levels (5 mg/L; ref. <5) and elevated D-dimer levels >35000 Ug/L FEU (ref. 0–500) and low C4 0.04 g/L (ref. 0.1–0.4). Other blood workup was unremarkable: serum amylase 52U/L (ref. 30–110), lipase 9 U/L (0–160), LDH (s) 128 U/L (130–241), AST 24U/L (11–38), ALT 39 U/L (12–48), ALP 52 U/L (20–140), urea 1.6 mmol/L (2.8–8.3), creatinine (s) 49 umol/L (64–104), total bilirubin 11 umol/L (3-20), erythrocytes 4.31 × 1012/L (4.35–5.72), haemoglobin 131 g/L (138-175), thromocytes 189 × 109/L (158–424). Urine culture was sterile, EBV, CMV, Toxoplasma gondii serology revealed past contact.

Two hours after Cinryze® treatment, her symptoms resolved. On the next day, leukocyte levels were normal, and D-dimer values were significantly lower (4992 ug/L).

She was discharged from the hospital with Cinryze® i.v. as on-demand therapy and application of low weight molecular heparin s.c. (dalteparin 7500 IU s.c. once daily) was recommended through the entire pregnancy due to history of previous spontaneous abortion and elevated D-dimers.

After that episode, the attacks were more frequent, with abdominal angioedema attacks every 3 days, and peripheral edema with the swelling of hands almost every other day. At 17 weeks gestation, the patient was started on Cinryze® prophylactic therapy 1000 IU i.v., two times a week. As the pregnancy progressed, her attacks became more frequent despite prophylactic treatment, so during the final month of the pregnancy, she received Cinryze® every two to three days.

In January 2019, she gave birth to a male term infant (gestational age 38 weeks), via C-section. The newborn infant was eutrophic (birth weight 3370 g), and had an average head circumference (33 cm). Upon birth, the infant was mildly dyspneic, had regular heart rate (>120/min), pink skin colour, was mildly hypotonic and had normal reflexes. Apgar scores were 8 at 1ʹ and 8 at 5ʹ. Due to transitory tachypnea of the newborn, he required non-invasive respiratory support with heated and humidified high flow nasal cannula (HFNC) for six hours. The hospitalization was otherwise uneventful, and he was discharged after 8 days. Head ultrasound was normal; there were no signs of perinatal infection.

Before the C-section, the patient received Cinryze® 1000 IU iv. For the next two weeks after the delivery, prophylactic therapy was continued, and subsequently, icatibant as on-demand therapy was recommended. The patient chose not to breastfeed. After the delivery, the patient was symptom-free for 6 months and required no treatment for HAE. Her first abdominal attack after the symptom-free period was less intensive than during pregnancy. Overall, during the pregnancy, the patient in total received Cinryze® (1000 IU i.v.) 48 times.

## Discussion

In the report we presented a female patient with HAE type I, with pregnancy as an exact trigger for HAE attacks, leading to the progression of the number of attacks as the pregnancy advanced, and the patient required prophylactic treatment. Interestingly, attacks started in the early pregnancy with a severe presentation of the disease that was not previously present, and resolved immediately after the delivery, leaving the patient symptom free for several months. There are only a few case reports and studies available, which give a detailed description of HAE worsening during the pregnancy, but to our knowledge, non showed such progression despite prophylactic therapy [5-9].

Hereditary angioedema is a rare disease that affects both male and female patients, but the attacks are more frequent in female patients [10]. The likely explanation is the possible effect of estrogen on the kallikrein-kinin system and subsequently on the disease course. [11]. Furthermore, it is known that HAE attacks can be precipitated by estrogen replacement therapy and contraceptive hormones [4, 10].

In our patient, the first symptom appeared during puberty onset. Although the SERPING 1 mutation and low levels of C1 inhibitor and C4 complement were  found in her father and brother, she was the only member of the family with HAE symptoms.

The impact of pregnancy on HAE symptoms in patients is still unclear. Martinez-Saguer et al. showed that in 83% of pregnancies, attack rates increased during pregnancy with the highest rates in the second and third trimesters [12]. Czaller et al. in their study reported that attack frequency increases in 48% of pregnancies, most of them experienced worsening in the third trimester. Still, in 19% of the pregnancies, there was no influence on the course of HAE [9]. In contrast to these findings, Chinniah showed that women with HAE have significantly reduced or absent attacks in the last two trimesters of pregnancy [13].

In our patient, the first HAE symptom, when the pregnancy was revealed, was life-threatening laryngeal edema, whereas, before pregnancy, HAE symptoms were always presented as peripheral or abdominal edema. As the pregnancy progressed, her attacks became more frequent, especially in the last trimester, and were presented as abdominal and peripheral edema. Following the international WAO/EAACI guideline for the management of hereditary angioedema, the patient was treated with C1 inhibitor concentrate as prophylactic therapy during the pregnancy [1, 14].

Some authors suggest an increased number of attacks following frequent treatments with C1 concentrate [15]. This was not confirmed in our case, since our patient received 48 times pdC1 inhibitor concentrate and the attacks stopped after the delivery, and afterwards, she was a symptom-free for 6 months.

The patient was treated with pdC1 inhibitor concentrate Cinryze®, although there is no relevant epidemiological data regarding its safety during pregnancy. Until now, there are only a few case reports of its safety during pregnancy [16].

## Conclusion

In the case presented, the angioedema attacks worsened as the pregnancy progressed. After delivery, the patient was symptom-free for six months. During the pregnancy, she was treated with pdC1 inhibitor concentrate Cinryze®, which showed to be a safe choice, although there is no relevant epidemiological data regarding its safety during pregnancy. Our report further confirms that Cinryze® therapy is effective and safe treatment during pregnancy, with no adverse effects on the infant.

## Data Availability

Not applicable.
